# A231 SINGLE GUIDEWIRE MAIN PANCREATIC DUCT CANNULATION AS A RISK FACTOR FOR POST-ERCP PANCREATITIS: FINDINGS FROM A MULTI-CENTER PROSPECTIVE ENDOSCOPY REGISTRY

**DOI:** 10.1093/jcag/gwae059.231

**Published:** 2025-02-10

**Authors:** M Gupta, M Chau, M Howarth, S Cartwright, S Ficaccio, B J Elmunzer, N Forbes

**Affiliations:** University of Calgary Cumming School of Medicine, Calgary, AB, Canada; Division of Gastroenterology and Hepatology, University of Calgary, Calgary, AB, Canada; Division of Gastroenterology and Hepatology, University of Calgary, Calgary, AB, Canada; Division of Gastroenterology and Hepatology, University of Calgary, Calgary, AB, Canada; Division of Gastroenterology and Hepatology, University of Calgary, Calgary, AB, Canada; Division of Gastroenterology and Hepatology, Medical University of South Carolina, Charleston, SC; Division of Gastroenterology and Hepatology, University of Calgary, Calgary, AB, Canada

## Abstract

**Background:**

Post-endoscopic retrograde cholangiopancreatography (ERCP) pancreatitis (PEP) remains a relatively common adverse event. Though multiple cannulations of the main pancreatic duct (PD) are a known risk factor for PEP, the impact of a single cannulation of the main PD remains controversial.

**Aims:**

Given the importance of identifying those at risk for PEP, especially in light of the growing evidence for preventative interventions such as PD stenting, we aimed to identify if single PD cannulation is a risk factor for PEP.

**Methods:**

We analyzed data from a multi-center, prospective registry collected from 2018-2024 with associated protocolized 30-day follow-up for patients undergoing ERCP for any biliary indication. PEP was defined as recurrent admission or prolongation of existing admission due to the development of characteristic abdominal pain occurring after ERCP with pancreatic enzyme elevation >3 times the upper limit of normal at least 1-day post ERCP and/or imaging findings characteristic of pancreatitis. The association of single PD cannulation and PEP was evaluated with multivariable logistic regression utilizing known risk factors and preventive interventions as covariates. Results were reported as odds ratios (OR) with associated 95% confidence intervals.

**Results:**

PEP complicated 287 (3.8%) of the 7,479 ERCPs studied. Single main PD cannulation (OR 2.14, 1.45-3.12), and multiple main PD cannulation (OR 2.58,1.76-3.76), were independent risk factors for PEP in all comers. This trend was also maintained in those with native papilla for single PD (OR 2.20, 1.43-3.24) and multiple PD (OR 2.50, 1.65-3.77) cannulations. Single main PD cannulation to both the pancreatic head (OR 1.89, 1.02-3.30) and the pancreatic body (OR 2.61, 1.44-4.57) were associated with PEP compared to no main PD cannulation. Further, a trend towards increased risk with any number of side PD cannulations (OR 1.15, 0.63-2.00) was seen, though these analyses were likely underpowered.

**Conclusions:**

Single main PD duct canulation, to the pancreatic head or body, represents an independent risk factor for PEP in this analysis. Additionally, there appears to be additive risk associated with multiple main PD cannulations and deeper main PD cannulations. These results highlight the risk of any degree of PD cannulation, supporting the potential utility of routine preventative interventions, such as PD stenting in these cases.

**Funding Agencies:**

None

